# Death Certification and Registration

**Published:** 1907-05-04

**Authors:** 


### The Hospital

A Journal of

The Medical Sciences and Hospital Administration.

New Series. No. 5, Vol. I. [No. 1077, Vol. XLII.] SATURDAY, MAY 4, 1907.

### Death Certification and Registration.

The main facts recently presented to the Lord Chancellor, by a deputation which represented the London County Council and the Medico-Legal Society, carry a fairly familiar aspect to members of the medical profession. It is no new thing to the general practitioner to hear that the existing machinery for the certification and registration of deaths is unsatisfactory. Nor will he be disposed to question the proposition that in many respects the legal inquiries that are called for in the case of uncertified deaths are open to much improvement. In his personal experience the practitioner of medicine is reminded only too frequently of an arrangement under which he is compelled by the State, under the threat of penalties, to perform a responsible duty without any suggestion either of recognition or reward. To refuse to grant a certificate of death, without definite and adequate reason, means for the medical man concerned the risk of police court proceedings, the payment of a fine, and more or less of public obloquy. Yet the authority which demands this service decrees that no acknowledgment shall be rendered for its discharge. Further, it is well known, and the statutory certificate indeed provides for the possibility, that the fact of death may be outside the immediate knowledge of the practitioner. The suggestion now advanced is that this shall be altered, and that a death certificate shall include a statement to the effect that the death of the individual in question has undoubtedly taken place. Everyone will agree that this is an absolutely essential proceeding, but if it is proposed that this shall be one more gratuitous service to be rendered by the profession to the State, we hope the profession will arrange an active opposition to the plan. Again, in connection with uncertified deaths many medical practitioners have been in the habit of giving information to coroners in order to help in the decision whether, in an individual instance, an inquest is or is not necessary. The law does not recognise such service, and in some quarters, what has been done out of courtesy and goodwill, has come to be claimed as a duty, the fulfilment of which may be demanded by a so-called "coroner's officer," to whose incompetent hands inquiries preliminary to an inquest are usually delegated. The suggestion now is that all such inquiries shall be conducted by specially appointed "medical investigators," and in so far as this means the abolition of the present stupid and blundering plan, it is worthy of sympathy. But it has still to be asked of whom these investigators are to inquire, and if the proposal means the creation of a sort of sub-coroner, who will have the right to demand from medical practitioners (among others) gratuitous and informal evidence, the guiding organisations of the profession should promptly take note of the suggestion.

In writing as above we are by no means unconscious of the large public motives which animate the proposals submitted to the Lord Chancellor by the representatives of the County Council and the Medico-Legal Society. The revelations made by the Death Certification Committee of 1893 exposed, as Sir William Collins rightly said, a public scandal, and though the facts have been pressed on the attention of successive Governments, they remain at the present day entirely without remedy. It is beyond question that the existing regulations leave room for the suspicion that cases of death from other than natural causes are occasionally passed by the registrars, and that, to take another instance, bodies are sometimes disposed of as still-born, which are, in fact, those of live-born children. Such a state of matters exhibits a serious flaw in the apparatus necessary for the public safety. Equally, on most of the other criticisms submitted to the Lord Chancellor, we find ourselves in substantial agreement with the views of the deputation, and we are sure that the medical profession now, as in the past, will be heartily in favour of sound and thorough reform. No mere selfish or personal interests will, for a moment, be pressed in opposition to proposals calculated to secure the larger interests of public welfare. At the same time, it is necessary to recognise that services demanded from the medical profession ought to receive appropriate recognition. Reformers have too often forgotten this elementary proposition, and the profession has been anything but emphatic in urging its legitimate claims. Hence with the utmost goodwill towards an improved system of death registration, the profession will be wise to see that this is not allowed to add further demands to the unremunerated duties which medical practitioners already discharge.

